# Causes of microcephaly in human—theoretical considerations

**DOI:** 10.3389/fnins.2023.1306166

**Published:** 2023-11-23

**Authors:** Michael Heide, Wieland B. Huttner

**Affiliations:** ^1^German Primate Center, Leibniz Institute for Primate Research, Göttingen, Germany; ^2^Max Planck Institute of Molecular Cell Biology and Genetics, Dresden, Germany

**Keywords:** basal progenitors, cortical stem and progenitor cells, cell lineages, cell division, Zika virus

## Abstract

As is evident from the theme of the Research Topic “*Small Size, Big Problem: Understanding the Molecular Orchestra of Brain Development from Microcephaly*,” the pathomechanisms leading to mirocephaly in human are at best partially understood. As molecular cell biologists and developmental neurobiologists, we present here a treatise with theoretical considerations that systematically dissect possible causes of microcephaly, which we believe is timely. Our considerations address the cell types affected in microcephaly, that is, the cortical stem and progenitor cells as well as the neurons and macroglial cell generated therefrom. We discuss issues such as progenitor cell types, cell lineages, modes of cell division, cell proliferation and cell survival. We support our theoretical considerations by discussing selected examples of factual cases of microcephaly, in order to point out that there is a much larger range of possible pathomechanisms leading to microcephaly in human than currently known.

## 1 Introduction

Microcephaly in human is a developmental disorder that results in a reduction of head size such that head circumference is three standard deviations lower than the mean for a human of the same age and sex. A major cause of microcephaly is an impaired development of the brain (microencephaly) (Kaindl et al., 2010; Gilmore and Walsh, 2013; Alcantara and O'Driscoll, 2014; Jayaraman et al., 2018; Phan and Holland, 2021; Zaqout and Kaindl, 2022), which is the focus of this treatise. Human individuals with microencephaly, whose brain size may be as small as that of a chimpanzee, our closest living relative, typically exhibit severe intellectual disability (Kaindl et al., 2010; Lui et al., 2011; Zaqout and Kaindl, 2022). This supports the notion that the expansion of the brain, notably of the neocortex, in the course of human evolution is one basis—though clearly not the only one—for the cognitive abilities that make us human (Rakic, 2009). The latter qualifying statement reflects the fact that the intellectual abilities of human individuals with severe microencephaly, albeit reduced, are still much greater than those of chimpanzees.

Understanding the causes that underlie human microencephaly is a fundamental challenge, as it is key for an early diagnosis of this neurodevelopmental disorder and, potentially, for appropriate therapeutic approaches. Here, we present a number of theoretical considerations dissecting proven, as well as possible, causes of human microencephaly as detected at birth.

**Figure 1:**
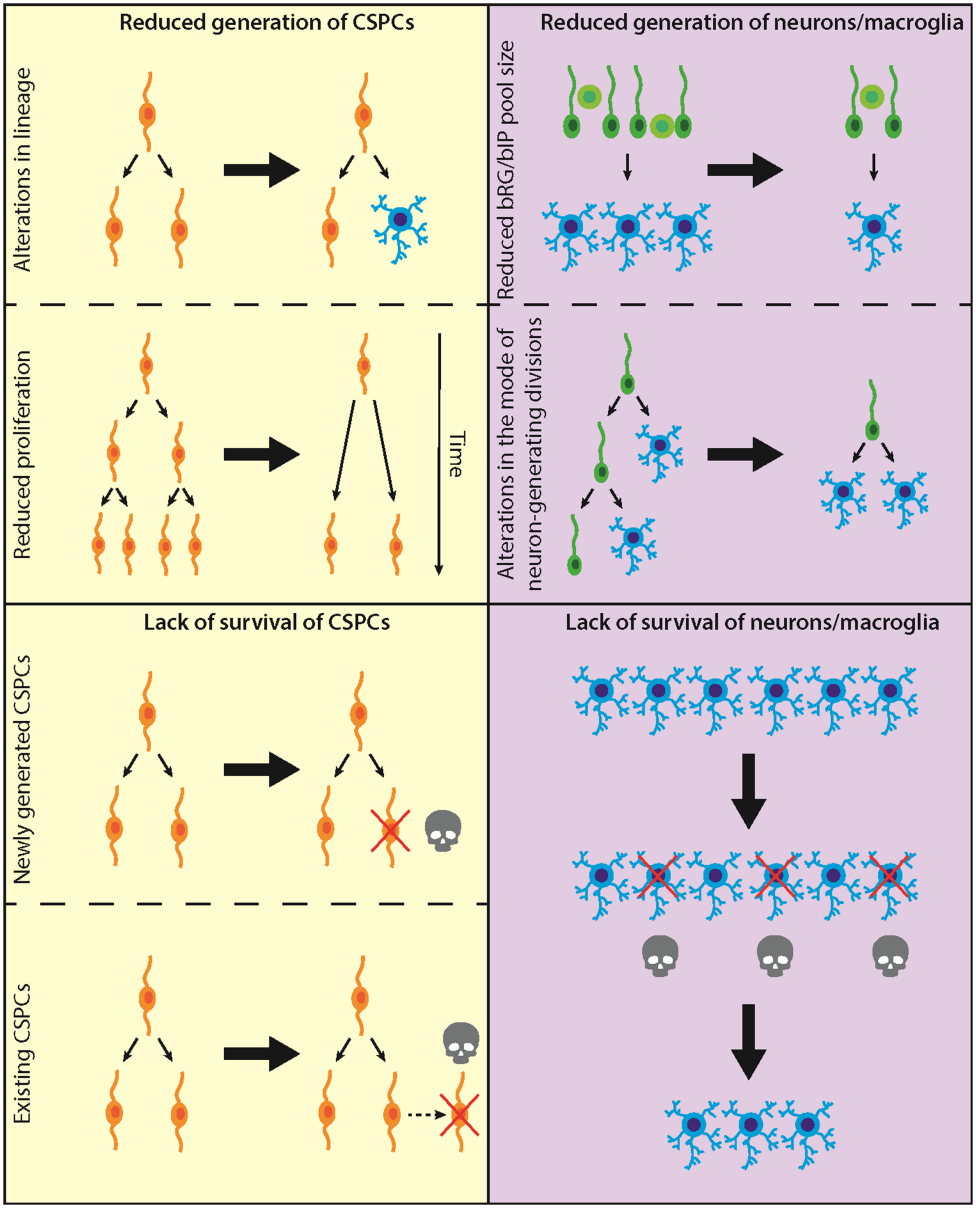
Diagram illustrating the proposed/established causes leading to microencephaly. Left: Causes at the level of CSPCs; top, CSPC generation; bottom, CSPC survival. Right: Causes at the level of neurons/macroglia; top, neuron/macroglia generation; bottom, neuron/macroglia survival.

Our treatise focuses on the development of the neocortex and comprises two classes of theoretical considerations, addressing (i) a reduced generation of cortical stem and progenitor cells (CSPCs), neurons, and/or macroglial cells (astrocytes, oligodendrocytes); and (ii) a reduced survival of newly generated CSPCs, neurons, and/or macroglial cells (see Figure 1 for a summary).

## 2 Reduced generation of CSPCs, neurons, and/or macroglial cells

### 2.1 Reduced generation of CSPCs

There are two classes of CSPCs in the developing neocortex: apical progenitors (APs), whose cell bodies reside in the primary germinal layer, the ventricular zone (VZ); and basal progenitors (BPs), whose cell bodies reside in the secondary germinal layer, the subventricular zone (SVZ) (Taverna et al., 2014). As first uncovered for the developing neocortex of primates (Smart et al., 2002), an inner SVZ (iSVZ), a zone adjacent to the VZ, and an outer SVZ (oSVZ), a zone located basally to the iSVZ, can be distinguished, with the oSVZ being particularly expanded in human. Each class of CSPCs comprises distinct types of CSPCs. Thus, APs comprise neuroepithelial cells (NECs), the primary stem cells of the brain, which with the onset of cortical neurogenesis transform into apical radial glia (aRG, also called ventricular radial glia) (Kriegstein and Götz, 2003; Taverna et al., 2014). Later in cortical development, aRG become truncated aRG (Nowakowski et al., 2016). A third type of AP are the apical intermediate progenitors (aIPs), previously called short neural precursors (Taverna et al., 2014). BPs comprise basal radial glia (bRG, also called outer radial glia) and basal intermediate progenitor cells (bIPs) (Taverna et al., 2014). Cellular and molecular features of these various CSPC types that are relevant for the development of microencephaly will be discussed in the respective sections below.

There are two principal causes underlying a reduced generation of CSPCs, both of which result in a decreased abundance of specific types of CSPCs, or even of all CSPCs. First, alterations in the physiological lineages leading to the various types of CSPCs (Figure 1, top left). Second, a reduction in the proliferative capacity of specific CSPC types (Figure 1, top left). Both causes underlying reduced CSPC abundance ultimately lead to a reduced generation of neurons and/or macroglial cells. As the latter two classes of neural cells account for most of the mass of the developed neocortex, their generation therefore is key for preventing the development of microencephaly.

#### 2.1.1 Alterations in CSPC lineages

**Figure 2:**
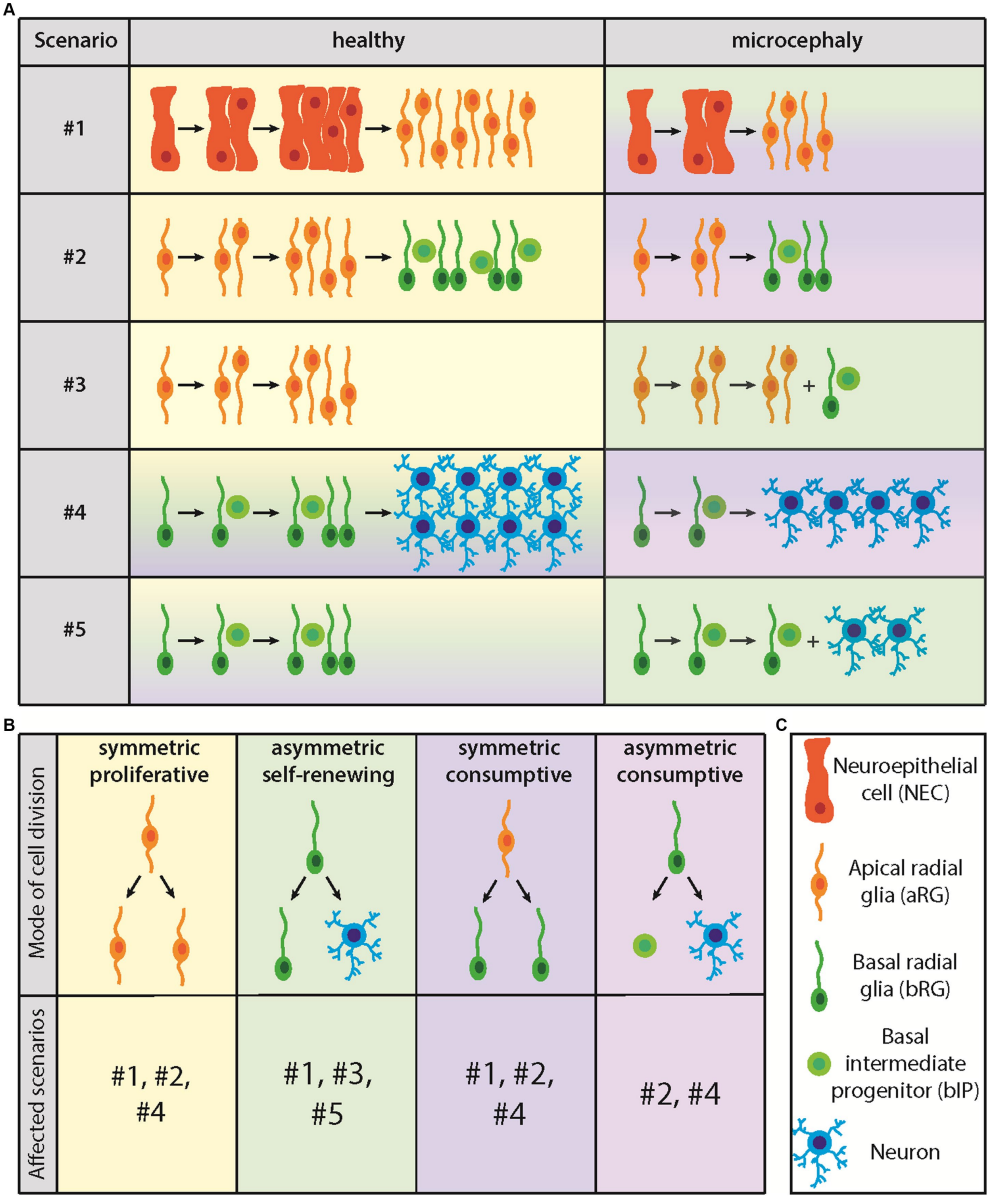
Diagram illustrating the five proposed CSPC lineage scenarios leading to microcephaly and the four modes of CSPC division. **(A)** Lineage scenarios leading to microcephaly. For details, see text. **(B)** Modes of CSPC division and lineage scenarios concerned. **(C)** Cell types involved.

The canonical overall CSPC lineage is: APs make more APs make BPs make more BPs, with the BPs then generating neurons and macroglial cells (Götz and Huttner, 2005; Kriegstein et al., 2006) (see Figure 2C for cell types). Within this overall CSPC lineage, the various types of APs and BPs need to be considered (Taverna et al., 2014). Alterations in any of these three major CSPC lineage steps, or regarding the differential roles of the various types of APs and BPs in the overall CSPC lineage, can be detrimental for the abundance of the relevant type of BP and hence for the appropriate generation of neurons and/or macroglial cells in developing human neocortex. For example, at the level of NECs, it has been shown that the transition to aRG is delayed in human as compared to other great apes (Benito-Kwiecinski et al., 2021). This delay is thought to underlie the greater NEC founder pool size in developing forebrain of human as compared to other great apes, a first step toward the evolutionary expansion of the human neocortex. It follows from this that an impairment of the delay of NEC-to-aRG transition would ultimately result in smaller pool sizes of the CSPC progeny of NECs, and hence could cause microencephaly (Figure 2A, scenario #1).

In addition to the above example of NEC-to-aRG transition (scenario #1) (Kriegstein and Götz, 2003; Götz and Huttner, 2005), and generalizing this issue, the following alterations in the overall CSPC lineage, and regarding the differential roles of the various types of APs and BPs therein, could result in a reduced abundance of the relevant BP type required for neocortex expansion during fetal development. These alterations, which are not mutually exclusive and probably constitute an incomplete set of possible scenarios, should therefore be considered as potential causes leading to human microencephaly. First, a reduction in aRG self-amplification, as this would reduce the aRG pool size and consequently that of BPs (Figure 2A, scenario #2) (Götz and Huttner, 2005; Kriegstein et al., 2006). Second, a premature onset of BP generation from aRG, as this would imply that the aRG pool does not reach its full size and, consequently, that the generation of BPs is lower than normal (Figure 2A, scenario #3) (Götz and Huttner, 2005; Kriegstein et al., 2006). Third, a reduction in BP self-amplification, pertaining to bRG and/or bIPs, as this would reduce the abundance of bRG and/or bIPs required for neocortex expansion during fetal development (Figure 2A, scenario #4) (Götz and Huttner, 2005; Kriegstein et al., 2006). Fourth, a premature onset of neuron and/or macroglia generation from BPs, as this would imply that the BP pool does not reach its full size and, consequently, that the generation of neurons and/or macroglia is lower than normal (Figure 2A, scenario #5) (Götz and Huttner, 2005; Kriegstein et al., 2006).

In all of the above 5 scenarios, the underlying cause of the alterations in CSPC lineages is a change in the mode of cell division of the AP or BP type concerned. This is therefore the next topic of our considerations.

##### 2.1.1.1 Changes in the mode of cell division of a given CSPC type

There are four modes of cell division that any type of CSPC can undergo and that are affected in the above 5 scenarios, (i) symmetric proliferative, (ii) asymmetric self-renewing, (iii) symmetric consumptive, and (iv) asymmetric consumptive (Figure 2B) (Taverna et al., 2014). In a symmetric proliferative division, a given CSPC type divides to generate two daughters of the same CSPC type as the mother CSPC, resulting in the self-amplification of this CSPC type (Taverna et al., 2014). This mode of CSPC division could be reduced in scenario #1 (premature NEC-to-aRG transition) and would be reduced in scenarios #2 (reduced aRG self-amplification) and #4 (reduced bRG and/or bIP self-amplification) (Figures 2A,B). In an asymmetric self-renewing division, a given CSPC type divides to generate one daughter of the same CSPC type as the mother CSPC, and another daughter that is a different type of CSPC or a non-CSPC (i.e., neuron or macroglial cell). This would prevent the self-amplification of the mother CSPC type, result in the maintenance of its pool size, and lead to the generation of a downstream CSPC type or non-CSPC (Taverna et al., 2014). Importantly, this mode of cell division would be repeatable. Switching to this mode of CSPC division could be a cause for the premature onset scenario #1 (premature NEC-to-aRG transition), and would underlie scenarios #3 (premature onset of BP generation from aRG) and #5 (premature onset of neuron and/or macroglia generation from BPs) (Figures 2A,B). Of note in this context, deletion of *Mcph1* in mice, which encodes microcephalin, results in microcephaly because of a premature switch from symmetric to asymmetric division of CSPCs (Zhou et al., 2013).

In a symmetric consumptive division, a given CSPC type divides to generate two equal daughters that are either of a different CSPC type or a non-CSPC (Figure 2B). This would result in the exhaustion of the mother CSPC pool and also lead to the generation of a downstream CSPC type or non-CSPC (Taverna et al., 2014). Importantly, however, this mode of cell division would be a single event for the CSPC concerned and would not be repeatable. Switching to this mode of CSPC division could also be a cause for the premature onset scenario #1 (premature NEC-to-aRG transition) (Figures 2A,B). In addition, switching to this mode of CSPC division could underlie the reduction in symmetric proliferative divisions in scenarios #2 (reduced aRG self-amplification) and #4 (reduced bRG and/or bIP self-amplification) (Figures 2A,B). Otherwise, this mode of CSPC division applies to the end of neurogenesis and/or macrogliogenesis, when the pools of aRG and BPs shrink. Finally, in an asymmetric consumptive division, a given CSPC type divides to generate two unequal daughters that are either of different CSPC types or non-CSPCs (i.e., neuron plus macroglial cell) (Figure 2B). This would also result in the exhaustion of the mother CSPC pool and lead to the generation of either downstream CSPC types or non-CSPCs (Taverna et al., 2014). Again, this mode of cell division would be a single event for the CSPC concerned and would not be repeatable. Switching to this mode of CSPC division could also underlie the reduction in symmetric proliferative divisions in scenarios #2 (reduced aRG self-amplification) and #4 (reduced bRG and/or bIP self-amplification) (Figures 2A,B). Otherwise, this mode of CSPC division also applies to the end of neurogenesis and/or macrogliogenesis, when the pools of aRG and BPs shrink.

We have described these possible changes in the modes of CSPC division in such detail in order to emphasize that at the level of CSPC division, there a many potential causes leading to microencephaly. A challenge of future research will be to explore if any of the existing forms of microencephaly in human with unknown etiology is caused by alterations in CSPC lineages and changes in the mode of CSPC division.

In this context, with regard to the modes of AP division, the orientation of the cleavage plane has been shown to have a central role (Taverna et al., 2014; Matsuzaki and Shitamukai, 2015). Thus, symmetric proliferative AP divisions require a vertical cleavage plane, i.e., a cleavage plane orientation parallel to the apical-basal axis of the AP, whereas oblique or horizontal cleavage planes, i.e., cleavage plane orientations deviating from the apical-basal axis of the AP, are associated with asymmetric self-renewing, symmetric consumptive or asymmetric consumptive AP division. All of latter three modes of AP divisions prevent APs from reaching their full pool size, which however is required for the growth of the human neocortex to its appropriate size during fetal development. The orientation of the cleavage plane is known to be perpendicular to that of the mitotic spindle. Hence, symmetric proliferative AP divisions require a horizontal orientation of the mitotic spindle, i.e., perpendicular to the apical-basal axis of the AP (Taverna et al., 2014; Matsuzaki and Shitamukai, 2015). Interestingly, in embryonic mouse neocortex, knockdown of *Aspm*, the mouse ortholog of human *ASPM* (*Abnormal Spindle-like Microcephaly-associated*), interferes with the maintenance of a horizontal mitotic spindle orientation after the onset of anaphase, shifting the mode of AP division from symmetric proliferative to asymmetric self-renewing, symmetric consumptive or asymmetric consumptive (Fish et al., 2006, 2008). Knockout of *Aspm* in ferret, a gyrencephalic carnivore, results in severe microcephaly (Johnson et al., 2018). Mutations in *ASPM* are the most common cause of autosomal recessive primary microcephaly in human (Bond et al., 2002; Kaindl et al., 2010; Phan and Holland, 2021; Zaqout and Kaindl, 2022). Hence, the change in AP cleavage plane orientation observed upon *Aspm* knockdown in embryonic mouse neocortex provides a striking mechanistic explanation how a change in the mode of AP division can lead to microencephaly.

#### 2.1.2 Reduced proliferation of a given CSPC type

As should be implicit from the discussion in the previous section, growth of the human neocortex to its appropriate size during fetal development requires that the relevant CSPCs, involved in ensuring the generation of the necessary number of neurons and macroglial cells, themselves are generated in sufficient numbers. This in turn requires an appropriate proliferative capacity of these CSPCs to self-amplify by symmetric proliferative divisions. In this section, we will discuss various scenarios where a reduction in CSPC proliferative capacity has been shown, or could well be, a cause of microencephaly (Figure 1, top left).

Reduced proliferation of a given CSPC type can be the result of various causes, both cell-intrinsic and cell-extrinsic. Major cell-intrinsic causes are changes in the modes of cell division, which may come about due to mutations in key genes, as has been discussed in the previous section, with *ASPM* as a prime example. Here, we will focus on cell-extrinsic causes of reduced CSPC proliferation. Classical examples of such causes are related to problems with growth factors that normally maintain the proliferative capacity of a certain type of CSPC or stimulate it. Such growth factors may act in (i) an autocrine manner, that is, being secreted from the same type of CSPC on which it acts; (ii) a paracrine manner, that is, being secreted from other types on CSPCs in the neighborhood; or (iii) an endocrine manner, that is, reaching the brain via the circulatory system after their production elsewhere in the body. Regarding, the latter, it is interesting to note that bIPs have been found to be often located in the vicinity of blood vessels (Javaherian and Kriegstein, 2009; Stubbs et al., 2009), suggesting the existence of a neurogenic niche for these CSPCs and raising the possibility that blood-derived growth factors may affect BP proliferation. In the following, we will discuss what may well be a paradigmatic example of an altered growth factor—receptor interaction being the cause of human microencephaly.

##### 2.1.2.1 Altered growth factor—receptor interaction

A well-known example for an altered growth factor—receptor interaction is the interaction between Insulin-like Growth Factor 1 (IGF-1) and Insulin-like Growth Factor 1 Receptor (IGF1R). In human, mutations in *IGF-1* and/or *IGF1R* lead to reduced binding of the IGF-1 ligand to the IGF1R receptor, resulting in microencephaly (Netchine et al., 2009; Gannage-Yared et al., 2013). Interestingly, mice overexpressing *IGF-1* exhibit an increase in brain size, which is accompanied by an increase in proliferating cells in the VZ and SVZ (Popken et al., 2004). This increase in proliferating cells is mediated by an accelerated cell cycle (shorter G1 phase) and increased cell cycle re-entry (Hodge et al., 2004). These data indicate that microencephaly in human associated with mutations in *IGF-1* and/or *IGF1R* are likely caused by reduced proliferation of CSPCs.

### 2.2 Reduced generation of neurons and/or macroglial cells from BPs

As already mentioned in the previous section, the overwhelming majority of neurons and macroglial cells in the developing human neocortex are generated from the two types of BPs, the bRG and the bIPs. There are two principal causes underlying a reduced generation of neurons and/or macroglial cells from BPs. First, a reduced pool size of either bRG, bIPs, or both types of BPs (Figure 1, top right). Second, a reduced number of neuron-generating divisions of either bRG, bIPs, or both types of BPs (Figure 1, top right).

#### 2.2.1 Reduced pool size of either bRG, bIPs, or both types of BPs

A reduction in the pool size of either bRG, bIPs, or both types of BPs (Figure 1, top right) can result from (i) alterations in the CSPC lineages due to changes in the modes of CSPC divisions that affect the abundance of either bRG, bIPs, or both types of BPs; or (ii) a reduced proliferative capacity of either bRG, bIPs, or both types of BPs; as discussed in the previous section. Regarding CSPC lineage, of relevance here is that aRG can generate both bRG and bIPs, and that bRG can generate bIPs (Taverna et al., 2014). Regarding proliferative capacity, both bRG and bIPs can self-amplify by symmetric proliferative divisions.

##### 2.2.1.1 CSPC lineage

As to changes in the lineage from aRG to bRG and/or bIPs and from bRG to bIPs, the reader is referred to the previous section in which the various scenarios of lineage alterations and of changes in the mode of CSPC division are discussed in detail.

##### 2.2.1.2 Proliferative capacity

As to a reduction in the proliferative capacity of either bRG, bIPs, or both types of BPs, both cell-intrinsic causes such as changes in the modes of cell division and cell-external causes such as problems with growth factors should be considered, as we have discussed in the previous section. However, there are certain aspects regarding the modes of neuron-generating divisions of bRG and bIPs that deserve further discussion.

#### 2.2.2 Differences in neuron-generating divisions between bRG and bIPs

There are two possible modes of neuron-generating divisions of a CSPC: (i) asymmetric self-renewing, where a CSPC self-renews itself and generates one neuron as the other daughter cell, and (ii) symmetric consumptive, where both daughter cells arising from a CSPC division are neurons, leading to the consumption of the CSPC (Taverna et al., 2014). Essentially all available data indicate that only the symmetric consumptive mode of neuron-generating division applies to bIPs. This reflects the lack of cell polarity of bIPs at mitosis (Taverna et al., 2014). In contrast, bRG maintain their intrinsic cell polarity throughout mitosis, as is evident from the fact that these CSPCs retain a basal process extending toward the basal lamina and/or an apically directed process at mitosis. This intrinsic cell polarity of bRG is the reason why the overwhelming majority of bRG divisions are asymmetric self-renewing (Taverna et al., 2014). The important implication of this difference is that bRG generate more neurons over time than bIPs (Fietz et al., 2010). Only toward the end of cortical neurogenesis are bRG thought to adopt the symmetric consumptive mode of neuron-generating division, which leads to the exhaustion of their pool. With regard to human microencephaly, specifically a reduction in cortical neurogenesis, a premature switch of bRG from the asymmetric self-renewing mode to the symmetric consumptive mode of neuron-generating division should therefore be considered as a possible cause (Figure 1, top right).

Although less data are available, the following considerations make it likely that similar scenarios apply, in principle, to a microencephaly that is associated with a reduction in the generation of macroglial cells. Thus, it is generally assumed that following neurogenesis, CSPCs switch to generate macroglial cells, first astrocytes and then oligodendrocytes (Malatesta et al., 2000; Qian et al., 2000). In fact, a seminal review on astrocyte development states: “*Multiple pathways that have been shown to play a role in gliogenesis may act by passively affecting the number of astrocytes by changing either the size of the progenitor pool or the timing of the neuron–glia switch*” (Molofsky et al., 2012). Consistent with this notion, the same transcription factor, Sox9, both increases BP proliferation and induces gliogenesis (Güven et al., 2020). A major part of astrogliogenesis takes place during the second half of fetal human brain development (Gilmore and Walsh, 2013). Taken together, these data are consistent with scenarios where a reduction in the CSPC pool size and/or a premature switch of gliogenic CSPCs to consumptive divisions may result in reduced macrogliogenesis and hence contribute to the generation to microcephaly at birth.

## 3 Reduced survival of newborn CSPCs, neurons, and/or macroglial cells

All scenarios discussed so far have addressed changes in the generation of CSPCs, neurons or macroglial cells. In this second part of our theoretical considerations, we will discuss intrinsic and extrinsic factors that affect the survival of cells, resulting in microencephaly. In principle, there are two different classes of cells the reduced survival of which may underlie microencephaly. First, lack of survival of CSPCs (Figure 1, bottom left). Second, lack of survival of terminally differentiated neural cells, i.e., neurons and macroglial cells (Figure 1, bottom right).

### 3.1 Lack of survival of CSPCs

There are two principal time points when CSPCs may die. CSPCs may die either directly after they have been generated, which is mainly caused by intrinsic factors (e.g., mutations in genes), or they may die at a later time point, which is mainly caused by extrinsic factors (e.g., toxins) (Figure 1, bottom left). In both cases, reduced survival of CSPCs would result in a reduced CSPC pool. This would lead to a diminished generation of neurons and/or macroglial cells and hence to microencephaly.

#### 3.1.1 Lack of survival of newly generated CSPCs

Death of newly generated CSPCs (Figure 1, bottom left) is normally a consequence of abnormalities during mitosis. For example, if a cell experiences prolonged mitosis, the mitotic surveillance pathway (MSP) gets activated and prevents the growth of the daughter cells by inducing apoptosis (Uetake and Sluder, 2010; Fong et al., 2016; Meitinger et al., 2016). Therefore, mitotic length is thought to be a readout for mitotic fidelity and cell health (Lambrus and Holland, 2017). While it is yet not completely understood how cells monitor mitotic length and activate the MSP, three of its key players have been identified, 53BP1, USP28, and P53. In this context, 53BP1 and USP28 appear to be upstream of P53, as knockout of these genes prevents stabilization of P53 in cells that experienced prolonged mitosis (Fong et al., 2016; Lambrus et al., 2016; Meitinger et al., 2016).

With regard to microencephaly, prolonged mitosis may be caused by mutations in genes which encode for proteins with functions at the centrosome or spindle apparatus (for an overview of such genes, see (Chavali et al., 2014)). For example, mutations in the gene encoding Centrosomal Protein 63 (*CEP63*) or in the gene encoding Centromere Protein J (*CENPJ*, also called *SAS4*) have been found to lead to microencephaly in human (Bond et al., 2005; Sir et al., 2011). In this context, *Cep63* and *Sas4* microcephalic mouse models show a prolonged mitosis of CSPCs in embryonic neocortex. This led to P53 activation and apoptosis in the progeny of CSPCs. Interestingly, these effects can be rescued by knockout of MSP components, resulting in reduced apoptosis which in turn restored the CSPC pool and led to normal brain size (Phan et al., 2021).

#### 3.1.2 Death of existing CSPCs

Death of existing CSPCs (Figure 1, bottom left) is normally due to extrinsic factors. Extrinsic factors can be either toxins (e.g., alcohol or drug abuse) or infectious agents (e.g., viruses, bacteria or protozoa), as is discussed in greater detail below. However, death of existing CSPCs can also be induced by intrinsic factors. One example for this is the reduced growth or even lack of blood vessels. At the beginning of neurogenesis, CSPCs are less depending on nutrient and oxygen supply from blood vessels. However, as soon as the CSPC pool expands, nutrient and oxygen get limited. Accordingly, during normal brain development, vascularization develops simultaneously with CSPC expansion (reviewed for example in Paredes et al., 2018). Mutations in genes regulating vascularization can have drastic effects on the survival of CSPCs (see below). As already mentioned above, death of existing CSPCs would result in a diminished CSPC pool, leading to a reduced generation of neurons and/or macroglial cells and hence to microencephaly.

##### 3.1.2.1 Toxins (alcohol, drug abuse)

Extrinsic factors, like toxins, can have dramatic effects on the existing CSPC populations. Prenatal exposure to alcohol, while easily preventable, is one of the main causes of neurodevelopmental disorders (Roozen et al., 2016). Fetal alcohol spectrum disorders encompass many different disorders which are caused by prenatal exposure to alcohol. These include the fetal alcohol syndrome (FAS), which is associated with microencephaly. A recent study (Adams et al., 2023) using brain organoids has provided insight into the pathomechanism underlying FAS. In this study, ethanol-exposed brain organoid cultures were smaller in size in comparison to control brain organoid cultures, mimicking the microencephaly observed in FAS. This reduction in brain organoid size in ethanol-exposed cultures was associated with a smaller population of Ki67-positive cells, i.e., CSPCs. These ethanol-treated organoids also showed a slightly altered cell cycle, with fewer cells in S-G2-M, in comparison to untreated organoids. However, the effect of this slightly altered cell cycle is most likely not sufficient to explain the strong reduction in cycling CSPC abundance. Rather, in addition to the altered cell cycle in ethanol-treated brain organoids, a strong increase in the number of apoptotic, caspase 3-positive cells was found in comparison to control brain organoids (Adams et al., 2023). This loss of CSPCs has a higher impact on the reduction of the CSPC pool and is likely the main underlying process leading to the microcephaly associated with FAS.

##### 3.1.2.2 Infections [viruses (Zika, CMV, HSV), bacteria (syphilis), protozoa (toxoplasmosis)]

Infections during pregnancy can have tremendous effects on fetal brain development. Different kinds of infectious agents can cause microcephaly, ranging from viruses (like Zika virus, cytomegalovirus, herpes simplex virus, rubella), to bacteria (syphilis) and protozoa (toxoplasmosis). Here, we focus on discussing the pathomechanism of the microencephaly due to Zika virus infection. A few years ago, during the Zika virus epidemic, microencephaly, which is caused by infection with this virus, became one focus of microcephaly research. In this context, brain organoids, which were developed 2 years earlier (Kadoshima et al., 2013; Lancaster et al., 2013), were used intensively. Similar to the extensive cell death detected in Zika virus-infected fetal human brains (Driggers et al., 2016), infected brain organoids showed increased caspase 3 activity and cell death in comparison to uninfected brain organoids, resulting in a smaller organoid size (Cugola et al., 2016; Qian et al., 2016; Li et al., 2017). In addition, mouse models also showed an increase in caspase 3-positive cells, resulting in a reduced CSPC pool and a smaller brain (Li et al., 2016; Miner et al., 2016; Shao et al., 2016). Further analyses in these mouse models and also in cultured CSPCs showed that Zika virus infection leads to activation of the innate immune response, notably of toll-like receptor (TLR) signaling (Dang et al., 2016; Wu et al., 2016; Zhang et al., 2016). This activation of TLR signaling led to CSPC apoptosis in brain organoids and impaired neurogenesis. Inhibition of TLR3 in Zika virus-infected brain organoids rescued the effect of the virus on CSPC apoptosis and (at least partially) restored the organoid size, while a TLR3 agonist mimicked the features of Zika virus infection in organoids (Dang et al., 2016). Similar to the FAS, Zika virus infection of brain organoids also led to reduced proliferation and a dysregulated cell cycle in CSPCs (Cugola et al., 2016; Qian et al., 2016). This was further confirmed by data from infected mouse embryos, which showed fewer mitotic cells in the VZ/SVZ (Li et al., 2016; Wu et al., 2016). These cells seemed to be arrested in G1, S, or G2, as the number of cells in M phase was significantly reduced (Li et al., 2016; Tang et al., 2016).

##### 3.1.2.3 Diminished or lacking vasculature in the developing brain

A prominent example of diminished vasculature in the developing brain is the Proliferative Vasculopathy and Hydranencephaly-Hydrocephaly (PVHH) Syndrome (also called Fowler Syndrome). This disorder is not only characterized by hydranencephaly–hydrocephaly and vasculopathy, but also microcephaly. Causative of Fowler Syndrome are mutations in the gene *FLVCR2* (Feline leukemia virus subgroup C cellular receptor family, member 2). Knockout of the mouse ortholog of *FLVCR2*, Major facilitator superfamily domain containing 7C (*Mfsd7c*), led to reduced blood vessel growth in the VZ and SVZ of the developing mouse brain. This resulted in hypoxia and cell death in the germinal zones, mainly in the VZ. The consequence of the reduced progenitor pool was a thinning of the cortex and microcephaly in *Mfsd7c* KO mice, suggesting a pathomechanism for the human patients affected by mutations in *FLVCR2* (Kalailingam et al., 2020).

### 3.2 Lack of survival of neurons and macroglial cells

Microencephaly in human may, of course, also be associated with impaired survival of neurons and/or macroglial cells (Figure 1, bottom right). In many cases, such impairment comes along with reduced survival of CSPSc. Thus, mutations in genes causing autosomal recessive primary microcephaly (MCPH) often lead to apoptotic death not only of CSPCs, but also of cortical neurons (Legiani et al., 2023). However, neuronal apoptosis can also be caused by mutations in genes expressed in, or specifically relevant for, neurons but not CSPCs. Paradigmatic examples of these are (i) mutations in *MAST1*, which encodes a microtubule-associated protein (Tripathy et al., 2018), and (ii) mutations in *SLC7A5*, encoding a large neutral amino acid transporter (Knaus et al., 2023). A somewhat intermediate situation, with apoptosis occurring predominantly in neurons but also—albeit to a lesser extent—in CSPCs, is the ligase 4 (LIG4) syndrome, where reduced LIG4 activity impairs nonhomologous end joining (NHEJ)-mediated DNA repair (Lun et al., 2019). These examples illustrate the spectrum of disorders that lead to neuronal apoptosis and thus result in microencephaly in human.

Regarding the survival of macroglial cells, not only CSPCs, but also astrocytes have been shown to be susceptible to Zika virus infection, which—as described above—causes microcephaly (Rubio-Hernandez et al., 2023). Moreover, Zika virus infection impairs proliferation and differentiation of oligodendrocyte progenitor cells and abolishes the development of oligodendrocytes (Li et al., 2018).

## 4 Conclusion

The theoretical considerations about possible causes of microencephaly that we have presented in this treatise make it evident that there likely exists a plethora of pathomechanisms leading to human microcephaly. This notion is supported by the data that the selected, factual cases of microcephaly we have discussed span a very broad spectrum of underlying causes. Thus, diverse cellular and molecular processes and targets, including but not confined to genes, may be subject to aberrations that result in microcephaly. It is hoped that the theoretical considerations presented here will help not only to provide insight into possible causes of human microcephaly, but also to suggest future avenues toward exploring potential therapeutic options.

## Data availability statement

The original contributions presented in the study are included in the article/supplementary material, further inquiries can be directed to the corresponding author.

## Author contributions

MH: Conceptualization, Writing – original draft, Writing – review & editing. WH: Conceptualization, Writing – original draft, Writing – review & editing.
